# Protective Effect of 18*β*-Glycyrrhetinic Acid against Triptolide-Induced Hepatotoxicity in Rats

**DOI:** 10.1155/2017/3470320

**Published:** 2017-05-09

**Authors:** Guanghua Yang, Lan Wang, Xiuting Yu, Yanfeng Huang, Chang Qu, Zhenbiao Zhang, Dandan Luo, Ji Lin, Lian Zhou, Ziren Su, Xiaojun Zhang, Haiming Chen

**Affiliations:** ^1^School of Chinese Materia Medica, Guangzhou University of Chinese Medicine, Guangzhou 510006, China; ^2^The First Affiliated Hospital of Chinese Medicine, Guangzhou University of Chinese Medicine, Guangzhou 510405, China; ^3^Guangdong Provincial Key Laboratory of New Chinese Medicinals Development and Research, Guangzhou University of Chinese Medicine, Guangzhou 510006, China; ^4^Dongguan Mathematical Engineering Academy of Chinese Medicine, Guangzhou University of Chinese Medicine, Dongguan 523808, China; ^5^Guangdong Provincial Hospital of Chinese Medicine, The Second Clinical College of Guangzhou University of Chinese Medicine, Guangzhou University of Chinese Medicine, Guangzhou 510120, China; ^6^Postdoctoral Programme, Guangzhou University of Chinese Medicine, Guangzhou 510120, China

## Abstract

Triptolide (TP) is the major active component of* Tripterygium wilfordii* Hook F (TWHF) and possesses multiple pharmacological effects. However, hepatotoxicity of TP which is one of the toxic properties slows its progression in clinical application. 18*β*-Glycyrrhetinic acid (GA) is the main bioactive ingredient of Licorice (*Glycyrrhiza glabra* L.), a herbal medicine famous for its detoxification. This study aims to investigate whether GA possesses protective effect against TP-induced hepatotoxicity in rats. TP interference markedly elevated serum levels of ALT, AST, and ALP, caused evident liver histopathological changes, and elevated hepatic TNF-*α*, IL-6, IL-1*β*, and IFN-*γ* as well as nuclear translocation of NF-*κ*B. TP also significantly elevated liver MDA and declined hepatic activities of SOD, CAT, and GSH-Px. Assay of TUNEL and apoptosis proteins (Bax, Bcl-2, and active caspase-3) showed that TP induced severe hepatocellular apoptosis. In contrast, low-dose GA (50 mg/kg) significantly reversed TP-induced changes above. However, high-dose GA (100 mg/kg) had no such effect. Overall, these findings indicated that low-dose GA but not high-dose GA exhibited a protective effect against TP-induced hepatotoxicity in rats by anti-inflammation, antioxidation, and antiapoptosis, which suggests that the doses of GA/Licorice should be carefully considered when used together with TWHF or TWHF preparations.

## 1. Introduction

**Figure 1:**
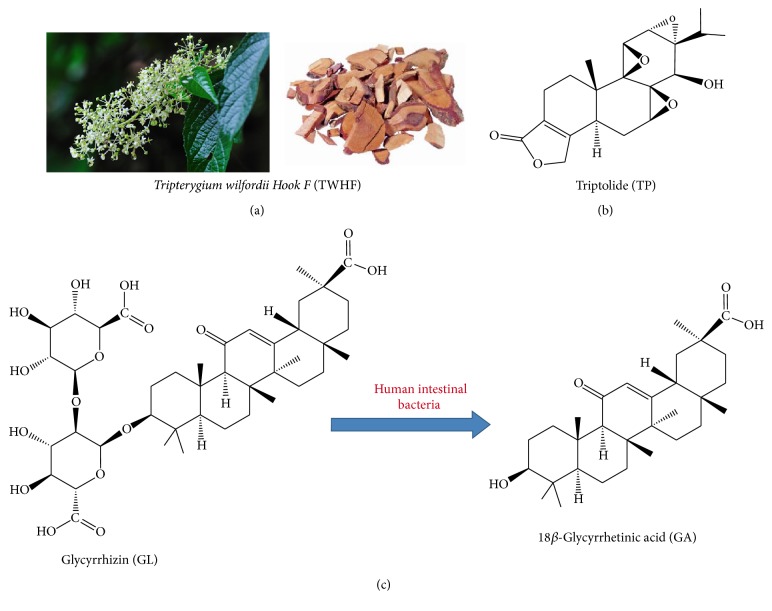
Image of* Tripterygium wilfordii *Hook F (a) and structure of triptolide, glycyrrhizin, and 18*β*-glycyrrhetinic acid (b, c).

*Tripterygium wilfordii* Hook F (TWHF), a woody climber known as Thunder God Vine, is abundant in eastern and southern China, Japan, and North Korea [1] (Figure 1(a)). The earliest account of its medicinal uses is in “Dian Nan Ben Cao” (Yunnan Materia Medica) in 1476 [2]. However, TWHF began to be of concern worldwide as its extracts exhibited high effectiveness in the treatment of leprosy and rheumatoid arthritis (RA) in numerous Chinese clinical reports from the 1960s [2]. Extracts of TWHF were again confirmed to be effective on RA by a clinical test (double-blind and placebo-controlled) in America [3]. In China, preparations of TWHF have been widely used to relieve RA, systemic lupus erythematosus (SLE), psoriasis, and immune complex nephritis and to prolong allograft survival in organ transplants [4]. Besides,* Tripterygium* glycoside (one extract of TWHF) has been included in China's national essential drugs [2].

The verified clinical efficacy of TWHF strongly stimulated the research of its bioactive ingredients [5]. Triptolide (TP), the first isolated diterpenoid triepoxide lactone from TWHF in 1972, possesses various promising pharmacological effects such as immunosuppression, anti-inflammation, and antitumor, which has been recognized as the main bioactive ingredient of TWHF with the related potencies 100~200 times higher than* Tripterygium* glycosides [1, 6] (Figure 1(b)). However, TP cannot be systemically used in clinic for its narrow therapeutic window and multiorgan toxicities [1, 7]. Among the adverse events of TP, clinical high proportion of hepatotoxicity is the primary cause of death and is greatly noticed by pharmacists and toxicologists [7].

Licorice (*Glycyrrhiza glabra* L.), a perennial herb widely grown in China, Japan, and Russia, is one of the most frequently used herbal medicines in the world [8]. In numerous TCM classic monographs, Licorice has been claimed to “resolve the hundred toxins,” for its detoxification property [9, 10]. Glycyrrhizin (GL), a major component of Licorice, has been widely used as an antidote in Asia and Europe [11]. GL is metabolized by the intestinal flora to 18*β*-glycyrrhetinic acid (GA) after oral administration, and GA is then absorbed into the blood [12, 13] (Figure 1(c)). Studies suggest that the biotransformation to GA possibly is the prerequisite for GL to exert its detoxification and other forms of pharmacological functions [14].

In Chinese clinical practices, Licorice is often used in combination with TWHF or TWHF preparations to reduce its adverse effect such as diarrhea and hepatic lesion [15, 16] and is always in small doses, but not large doses (3~10 g/d, not exceeding 30 g/d), for large doses will substantially reduce the effect of detoxification [17, 18].

Studies have reported that TP-induced hepatotoxicity can be attributed to lipid peroxidation and hepatic apoptosis [7, 19], while GA possesses anti-inflammation, antioxidation, and hepatoprotection activities [20, 21] and has recently been reported to protect HepG2 cells against TP-induced oxidative stress [22]. However, it is unknown whether GA has an in vivo protective effect against TP-induced hepatotoxicity, which is clearly worth exploring as Licorice is often used in combination with TWHF. In addition, just as aforementioned, whether the detoxification effect of GA depends on its dose is an interesting question.

Therefore, the aim of this study is to observe whether GA possesses a protective effect against TP-induced hepatotoxicity and the potential mechanisms of action. Besides, this study also preliminarily examined the pharmacological difference between low-dose and high-dose GA.

## 2. Materials and Methods

### 2.1. Animals and House Condition

Healthy Wistar rats (number 44005800002779, male, 200 ± 20 g) were obtained from the Laboratory Animal Services Center, Guangzhou University of Chinese Medicine. Animals were kept under controlled conditions (12-hour dark/light cycle, 22 ± 2°C, humidity 50 ± 5%) to acclimatize for 7 days before use and these conditions were kept throughout the experiment. Standard diet (chaws) and water were provided ad libitum and rat cages were kept stainless. All the animal experiments were approved and conformed to the principles of Laboratory Animal Care and Use Committee in Guangzhou University of Chinese Medicine.

### 2.2. Chemicals and Regents

TP and GA were supplied by Ci Yuan Biotechnology Co., Ltd. (Xi'an, China) and Sigma-Aldrich Co. LLC (St. Louis, MO, USA), respectively. The purities (>98%) of TP and GA were detected by HPLC. Commercial kits used to detect the MDA level, SOD, CAT, and GSH-Px activities were obtained from Nanjing Jiancheng Biotechnology (Nanjing, Jiangsu, China). ELISA detection kits for TNF-*α*, IFN-*γ*, IL-1*β*, and IL-6 levels were purchased from eBioscience, Inc. (San Diego, CA, USA). Antibodies specific for NF-*κ*B, I*κ*B*α*, phosphor-I*κ*B*α* (p-I*κ*B*α*), Bax, Bcl-2, and active caspase-3 were procured from Abcam plc. (Cambridge, UK). TUNEL and BCA detection kits were obtained from Roche (Mannheim, Germany) and Thermo Scientific, Inc. (Rockford, IL, USA), respectively. Manipulations of the kits above were following manuals of the manufacturers, and all other reagents as well as chemicals used in this research were of analytical grade.

### 2.3. Animal Experiments

The Wistar rats were divided into five groups with 10 individuals for each group randomly (Table 1). Animals in normal control (NC) group received distilled water for 6 days and 0.5% CMC-Na for the last 3 days. Rats in TP model group (TP), GA low-dose group (GAL+TP), and GA high-dose group (GAH+TP) received distilled water, GA (50 mg/kg,* p.o.*, dissolved in distilled water), or GA (100 mg/kg,* p.o.*, dissolved in distilled water) for consecutive 6 days, respectively, and liver injury was induced by TP (2.4 mg/kg,* p.o.*, suspended in 0.5% CMC-Na) for the last 3 days. Animals in the above three groups received TP 6 hours after distilled water or GA treatment on the last 3 days. The doses of GA range within 5~75 mg/kg for rats in the existing hepatic protection reports [11, 20, 23, 24], so we choose a most commonly used dose (50 mg/kg, equivalent to Licorice 18.52 g/d for human) as the low dose. In order to explore whether a high-dose GA that exceeded the common level still has a hepatic protective effect, we choose a dose (100 mg/kg, equivalent to Licorice 37.04 g/d for human) as the high dose. In addition, to observe whether high dose of GA influences hepatic function by itself, we set a GA high-dose control (GAH) group, in which rats received GA (100 mg/kg) only.

Twenty-four hours after the last dosing, blood samples were collected using capillaries from rats' orbital venous plexus. Then the animals were sacrificed and livers were removed immediately. Serum was prepared by centrifugation at 4°C, 3500 rpm for 15 min as routine method. The supernatants of 10% liver homogenate were obtained by centrifugation at 4°C, 12000 rpm for 10 min, and kept at −80°C until analysis.

### 2.4. Determination of Liver Enzymes

The levels of ALT, AST, and ALP in rat serum were analyzed using automated biochemical analyzer 7180 (Hitachi, Japan) to assess liver functions.

### 2.5. Measurement of Proinflammatory Cytokine in Liver Tissue

Hepatic levels of TNF-*α*, IFN-*γ*, IL-1*β*, and IL-6 were assayed with ELISA kits.

### 2.6. Detection of SOD, CAT, GSH-Px, and MDA

Activities of SOD, CAT, and GSH-Px, as well as MDA level in liver homogenate were detected using reagent kits.

### 2.7. TUNEL Assay

Test kits (Roche, American) of DNA fragmentation were used to detect hepatocyte apoptosis. Proteases were added to the paraffin sections after dewaxing and rehydrating and incubated at 37°C for 30 min. Then the slides were washed in PBS, permeabilized on ice for 5 min, and incubated with TUNEL reaction mixture at 37°C for 120 min, next with converter-POD added and incubated at 37°C for 30 min. Subsequently, the slides were washed in PBS, incubated with substrate solution for 20 min, and analyzed under light microscopy. The TUNEL-positive nuclei were staining as dark brown, intensive, and homogenous, and the apoptotic indexes were evaluated through 10 random fields for each slide.

### 2.8. Western-Blot Analysis

Western-blot assays were processed to evaluate the expressions of NF-*κ*B (p65) in cytoplasm and nucleus, p-I*κ*B*α*, I*κ*B*α*, and active caspase-3. Liver tissues were homogenized immediately after sacrifice to prepare the total protein, nuclear protein, and cytoplasmic protein using Nuclear-Cytoplasm Extraction Kit (Cell Signaling Technology, USA). Protein concentrations were detected by BCA kits (Thermo Scientific, Inc.), and Histone H3 (nuclear protein) and *β*-actin (cytoplasmic protein) were used as controls. After electrophoresis on 10% SDS-PAGE gels, the proteins were transferred onto the PVDF membrane. After being blocked with 5% skim milk, the membrane was incubated overnight with the primary antibody (Abcam, UK) at 4°C. Subsequently, the blots were washed with TBST, incubated with the secondary antibody at room temperature for 30 min, again washed with TBST, and developed with ECL Plus Western Blot Detection System (Amersham International plc., Buckinghamshire, UK).

### 2.9. Histopathological and Immunohistochemical (IHC) Examination

Liver portions (approximately 0.5 mm^3^) in the same position of left lobe were immediately excised and then fixed in 4% paraformaldehyde solution, embedded in paraffin, and sectioned at 5 *μ*m thickness. Samples were stained by hematoxylin-eosin (H&E) for routine histopathological examination.

For IHC examination, 5 *μ*m thick liver slices were deparaffinized and incubated with the first antibody against Bax or Bcl-2 overnight. After being washed with PBS for three times, the slides were incubated with the secondary antibody conjugated with HRP polymer. After positive color reaction with DAB, the slides were then counterstained with hematoxylin, dehydrated, and sealed with mounting medium. The cells stained with anti-Bax or Bcl-2 antibody (in cytoplasm or on nucleus) were counted as Bax or Bcl-2 intensity.

### 2.10. Statistical Analysis

All data were expressed as mean ± SD and analyzed using one-way ANOVA (SPSS 17.0, SPSS Inc., Chicago, IL, USA). *P* < 0.05 was considered to be statistically significant.

## 3. Results

### 3.1. GA Protected against Liver Injury Induced by TP

**Figure 2:**
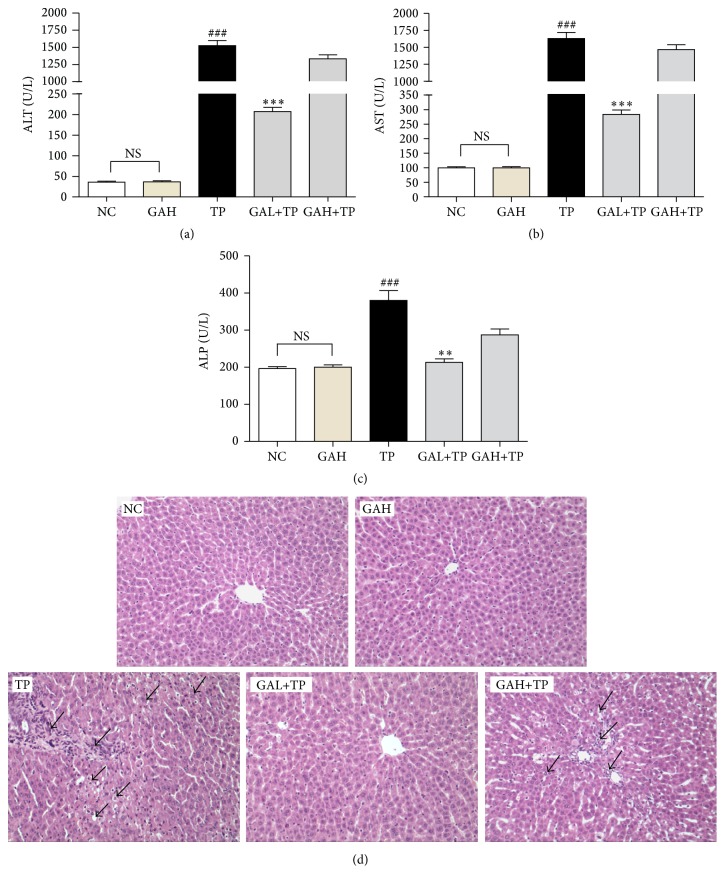
Effects of GA on TP-induced hepatic injury. Data were expressed as the mean ± SD (*n* = 10). ^###^*P* < 0.001 compared with the normal control group; ^*∗∗∗*^*P* < 0.001 and ^*∗∗*^*P* < 0.01 compared with the TP group; NS: no significance. H&E staining (d) was examined under a microscope (magnification, 200x). Arrows point to the lesion site.

Elevated serum ALT, AST, and ALP are the biochemical markers of hepatocellular damage. GAH group showed no observable differences compared with NC group, whereas, compared with NC group, TP group has significantly increased serum levels of ALT, AST, and ALP, indicating that TP* p.o.* at 2.4 mg/kg for three days induced severe liver injury. In contrast, rats in GAL+TP group which received low-dose GA (50 mg/kg) have significant reductions in the three serum parameters above when compared with TP rats. Although rats in GAH+TP group which received the high-dose GA (100 mg/kg) had slightly lowered the levels of three liver enzymes, the reductions did not reach statistical significance compared with TP group (Figures 2(a), 2(b), and 2(c)).

### 3.2. GA Alleviated the Histopathological Changes in Liver Induced by TP

In NC and GAH groups, hepatic lobular structures were clear and orderly, and hepatocytes with well-preserved cytoplasm and nucleus radially arranged around the central vein. On the contrary, the liver lobular structures were injured in TP group, with hepatocellular swelling/vacuolization obvious and inflammatory infiltration of massive leukocytes evident. Contrastingly, preadministration of low-dose GA protected animals from TP-induced hepatic lesions. The sections of GAL+TP group did not demonstrate obvious histopathological changes, which were almost comparable to those in NC and GAH groups. However, GAH+TP group still showed clearly structural disturbance and cellular damage, although it was slightly mitigated compared with TP group (Figure 2(d)). These histopathological observations were consistent with the above biochemical results of hepatic enzymes.

### 3.3. GA Inhibited Hepatic Proinflammatory Response Induced by TP

**Figure 3:**
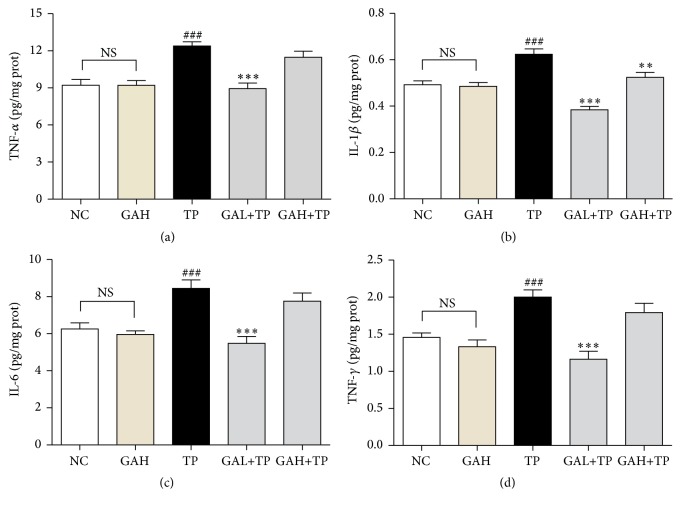
Cytokine changes in liver tissues. Data were expressed as the mean ± SD (*n* = 10). ^###^*P* < 0.001 compared with the normal control group; ^*∗∗∗*^*P* < 0.001 and ^*∗∗*^*P* < 0.01 compared with the TP group; NS: no significance.

Proinflammatory cytokines such as TNF-*α*, IL-1*β*, IL-6, and IFN-*γ* are mediators of inflammatory processes. TP treatment significantly increased hepatic TNF-*α*, IL-1*β*, IL-6, and IFN-*γ* protein levels, suggesting severe inflammatory response. On the contrary, low-dose GA (50 mg/kg) markedly suppressed the release of the four cytokines above. However, the high-dose GA (100 mg/kg) only suppressed IL-1*β* but did not change the levels of TNF-*α*, IL-6, and IFN-*γ* (Figure 3).

**Figure 4:**
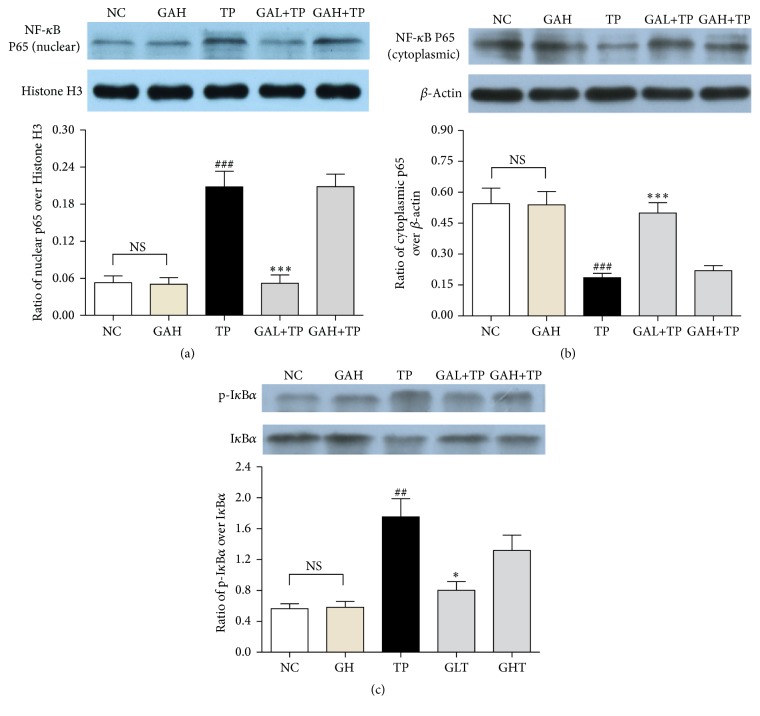
Effects of GA on liver NF-*κ*B activation. Data were expressed as the mean ± SD (*n* = 10). ^###^*P* < 0.001 and ^##^*P* < 0.01 compared with the normal control group; ^*∗∗∗*^*P* < 0.001 and ^*∗*^*P* < 0.05 compared with the TP group; NS: no significance.

NF-*κ*B is a central molecule in the inflammatory cascades, which translocates from cytoplasm to cell nucleus after activation to initiate transcription of inflammatory cytokines. To study whether the anti-inflammation of GA involved inhibition on NF-*κ*B activation, we measured the protein levels of NF-*κ*B (p65), p-I*κ*B*α*, and I*κ*B*α*. NF-*κ*B (p65) is expressed mostly in cytoplasm but not nucleus in NC and GAH groups. In contrast, in TP group, the NF-*κ*B (p65) expression in nucleus markedly increased. However, low-dose GA significantly inhibited the nuclear translocation of NF-*κ*B (p65). On the other hand, rats in TP group had significantly increased I*κ*B*α* phosphorylation, but low-dose GA pretreatment prevented this elevation. Yet, GA high-dose treatment did not affect both the activation of NF-*κ*B (p65) and phosphorylation of I*κ*B*α* (Figure 4).

### 3.4. GA Suppressed Hepatic Oxidative Stress Induced by TP

**Figure 5:**
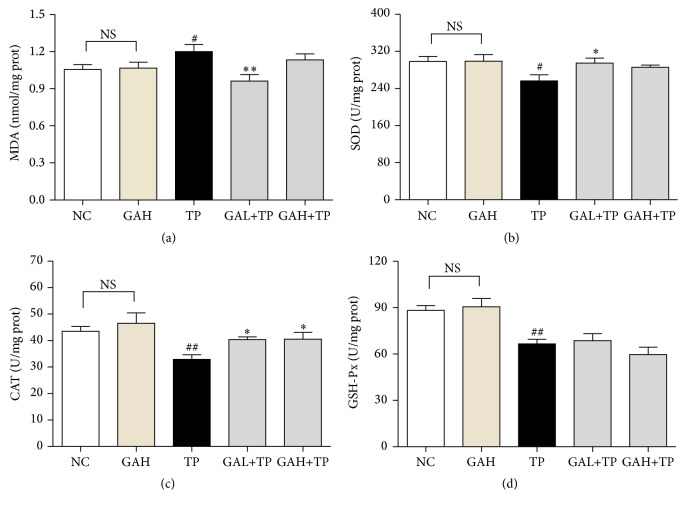
Effects of GA on TP-induced liver oxidative stress. Data were expressed as the mean ± SD (*n* = 10). ^#^*P* < 0.05 and ^##^*P* < 0.01 compared with the normal control group; ^*∗*^*P* < 0.05 and ^*∗∗*^*P* < 0.01 compared with the TP group; NS: no significance.

Oxidative stress has been considered to contribute to initiation and progression of liver injury. In order to evaluate whether GA protects rats against TP-induced oxidative stress, hepatic MDA level and the activities of SOD, CAT, and GSH-Px were measured. Hepatic MDA level was markedly elevated in TP group, which was significantly attenuated by low-dose GA, but not high-dose GA (Figure 5(a)).

On the other hand, significant reduction in the activities of SOD, CAT, and GSH-Px was found in TP rats' liver when compared with that in the NC group. In contrast, the activity of CAT was markedly increased by the two doses of GA treatments, and SOD activity was significantly enhanced by GA low-dose treatment. However, both doses of GA had no obvious influences on the activity of GSH-Px (Figures 5(b), 5(c), and 5(d)).

### 3.5. GA Protected Hepatocytes from TP-Induced Apoptosis

**Figure 6:**
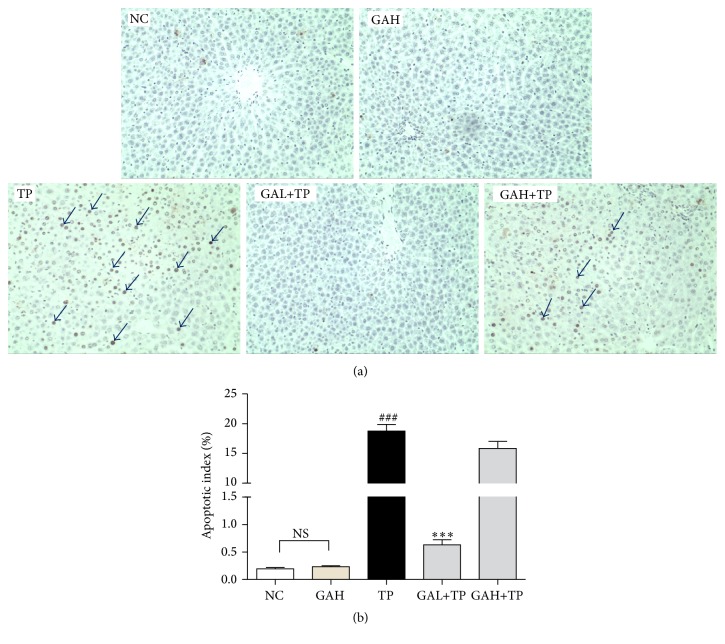
Effect of GA on TP-induced hepatocyte apoptosis. Data were expressed as the mean ± SD (*n* = 10). ^###^*P* < 0.001 compared with the normal control group; ^*∗∗∗*^*P* < 0.001 compared with the TP group; NS: no significance. (a) TUNEL stained liver sections (magnification, 200x). Arrows point to the positive cells.

Previous study had demonstrated that hepatocyte apoptosis was one of the mechanisms of TP-induced liver injury [19]. Therefore, we evaluated the effect of GA on TP-induced apoptosis. Sparsely distributed natural apoptosis of hepatocytes was viewed in NC and GH group. However, apoptosis was observed in all lobules with apoptotic index significantly increased in TP group. The GAL group had notably decreased apoptotic index compared with TP group, indicating that low-dose GA (50 mg/kg) protected hepatocytes from TP-induced apoptosis. However, high-dose GA (100 mg/kg) had no observable protection on the TP-induced hepatocellular apoptosis (Figure 6).

**Figure 7:**
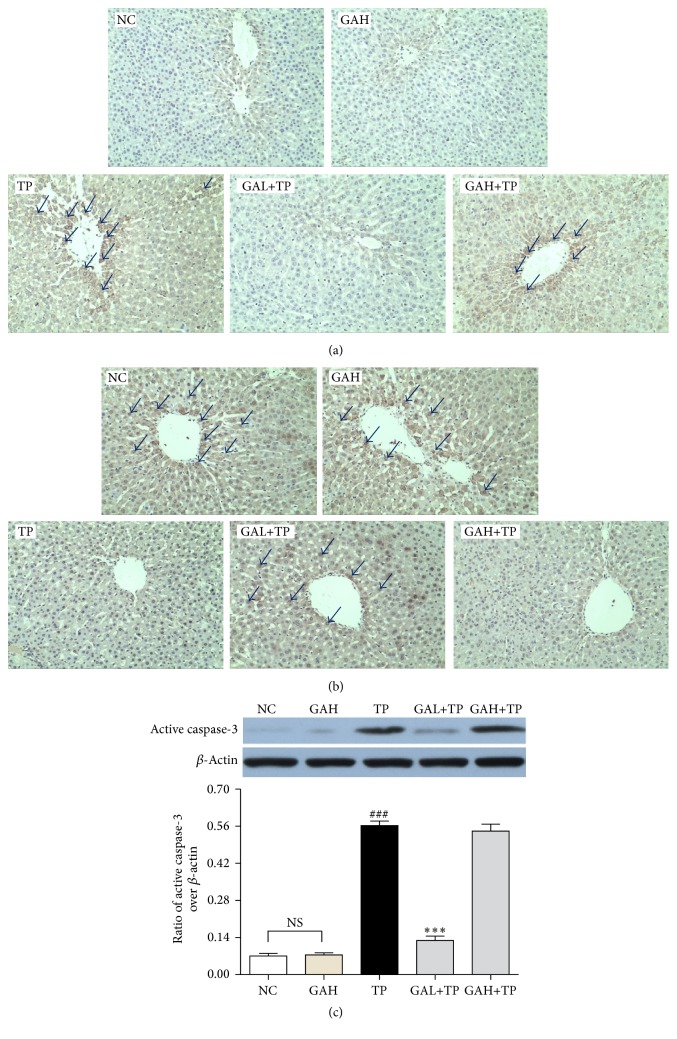
Effect of GA on Bax, Bcl-2, and active caspase-3. (a) IHC staining of Bax (magnification, 200x); (b) IHC staining of Bcl-2 (magnification, 200x); (c) Western-blot analysis of active caspase-3 protein in response to TP and GA. Data were expressed as the mean ± SD (*n* = 10). ^###^*P* < 0.001 compared with the normal control group; ^*∗∗∗*^*P* < 0.001 compared with the TP group; NS: no significance. Arrows point to the positive.

To further investigate the antiapoptotic mechanism of low-dose GA, we examined Bax/Bcl-2 protein expression through IHC staining and measured active caspase-3 protein level via Western-blot. TP group compared with NC group had elevated expression of Bax and declined expression of Bcl-2 and markedly high level of active caspase-3. However, these changes were all inhibited by low-dose GA, but not high-dose GA (Figure 7).

## 4. Discussion

As the main bioactive ingredient of TWHF, TP has been verified to possess inevitable anti-inflammatory and immunosuppressive activities in clinical and experimental researches (especially quite effective in the treatment of refractory autoimmune diseases such as RA, SLE, ankylosing spondylitis, and psoriasis) [25, 26] and can effectively prevent allograft rejection in organ transplants (including bone marrow, renal, cardiac, and skin transplants) [27]. Recent studies showed that TP can not only block tumor growth in vitro/in vivo directly, but also potentiate the antitumor effects of cytotoxic or chemotherapeutic agents [27, 28], which arouses wide concern on it. So, TP is of bright prospective in clinical applications and it is meaningful to find effective methods to reduce the toxicity of TP.

Our research demonstrated that GA protects rats against TP-induced hepatotoxicity at a low dose (50 mg/kg) but not high dose (100 mg/kg). The TP-induced hepatotoxicity is evident as rats in TP group had elevated serum levels of ALT, AST, and ALP, which are the biomarkers of liver diseases [29]. These enzymes are released into the blood circulation from the cytoplasm of hepatocytes following cell damage [30]. TP-induced liver injury was further confirmed by the histological alterations including damaged liver lobular structures, hepatocellular swelling/vacuolization, and inflammatory infiltration. In contrast, pretreatment with low-dose GA (50 mg/kg) showed a protective effect by maintaining structural integrity of liver tissue, significantly reducing the serum levels of ALT, AST, and ALP, and mitigating the liver histopathological changes.

During drug-induced hepatotoxicity, tissue damage leads immune cells and injured cells to generate inflammatory mediators, which in turn further promote tissue damage [31]. TP could activate Kupffer cells, which promote hepatic inflammation by producing TNF-*α*, IL-6, and IL-1*β* and thus accelerate the secretion of IFN-*γ* by NK cells [31, 32]. These proinflammatory cytokines can exacerbate drug-induced hepatocellular damage by amplifying the inflammatory process [31, 32]. In our study, treatment of TP did significantly increase the hepatic levels of TNF-*α*, IL-6, IL-1*β*, and IFN-*γ*, which demonstrated hepatic inflammation in rats, whereas pretreatment of low-dose GA downregulated the levels of the above proinflammatory cytokines, indicating the anti-inflammatory property of GA. This effect is consistent with the previous reports and is one of the significant mechanisms by which GA protected rats against TP-induced hepatotoxicity [20, 23].

As is well known that NF-*κ*B regulates multiple proinflammatory gene expression and plays a central role in inflammation [33], we further investigated NF-*κ*B signaling to explore the hepatic anti-inflammatory mechanism of GA. The activation of NF-*κ*B is closely related to the inhibitory protein I*κ*B. Inactive-state NF-*κ*B exists in cytoplasm and is bound to I*κ*B. Stimuli such as TNF-*α* and IL-1*β* lead to phosphorylation, ubiquitination, and degradation of I*κ*B by proteasomes, and then NF-*κ*B becomes active and translocates to nucleus where it activates various proinflammation related genes [34]. The results showed that GA low-dose treatment markedly restrained the cytoplasm-nucleus translocation of NF-*κ*B (p65) and increased I*κ*B*α* expression but markedly inhibited the increment of p-I*κ*B*α* in TP group. Therefore, these results suggest that low-dose GA mediated anti-inflammation in TP-injured liver possibly by inhibiting the transactivation of NF-*κ*B via stabilizing I*κ*B*α*.

On the other hand, TP-induced hepatotoxicity is also closely related to peroxidation caused by reactive oxygen species (ROS) [7]. TP brought about severe oxidative stress in rat livers as observed by markedly elevated MDA level and declined antioxidant enzyme activities (SOD, CAT, and GSH-Px), which is consistent with previous reports [7, 35]. MDA is one of the major secondary oxidation products, the content of which is an index of intensified peroxidation process [36]. The current results demonstrated that low-dose GA pretreatment significantly prevented the increase of MDA in TP rats, indicating that it greatly reduces lipid peroxidation.

SOD protects liver against spontaneous oxygen toxicity and lipid peroxidation by converting superoxide anions into H_2_O_2_ [37]. CAT protects cells from ROS injury by converting superoxide radicals (as well as H_2_O_2_) to molecular oxygen and water [7]. GSH-Px is another important enzyme for cells to clear ROS and protect against lipid peroxidation [7]. Our results showed that low-dose GA remarkably enhanced SOD/CAT activity in TP rats and thus strengthened the hepatic antioxidant defenses, although there was no significant elevation in GSH-Px activity.

Oxidative stress is an important trigger for cell apoptosis, and hepatocyte apoptosis is one of the mechanisms of TP-induced liver injury [19, 38]. GA could prevent apoptosis toward glycochenodeoxycholic acid-induced cytotoxicity in rat hepatocytes [39]. Therefore, we evaluated the effect of GA on TP-induced hepatocyte apoptosis and signaling proteins involved in mitochondrial apoptotic regulation. Caspase-3, which belongs to the family of cysteinyl aspartate proteases, is the primary effecter in cell apoptosis [40]. Proapoptotic protein Bax exists in cytoplasm and can translocate to outer mitochondrial membrane when stimulated to accelerate apoptosis. On the contrary, antiapoptotic protein Bcl-2 can restrain the effect of Bax [41]. In this study, TUNEL staining proved that the preadministration of low-dose GA significantly reduced the TP-induced apoptosis. IHC staining and Western-blot results revealed that low-dose GA pretreatment promoted Bcl-2 expression and inhibited the expression of active caspase-3/Bax. These data denoted that low-dose GA may mitigate TP-induced hepatocellular apoptosis via mediating apoptosis-related proteins. However, consistent with previous histological and biochemical results, high-dose GA showed no obvious regulation on these apoptotic signal proteins.

In TP-induced hepatotoxicity, oxidative stress is accompanied by the inhibition of Nrf2 [7]. GA has been reported to protect against cyclophosphamide-induced hepatotoxicity in rats through activation of Nrf2 pathway [20]. Nrf2 is anchored in cytoplasm in normal conditions. Oxidative stress causes Nrf2 to translocate from cytoplasm into nucleus, then activating multiple cytoprotective genes [7]. Thus, Nrf2 pathway is now considered as an important antioxidant signaling pathway, which is also involved in attenuating inflammation such as downregulating NF-*κ*B pathway and proinflammatory cytokines including TNF-*α*, IL-1*β*, and IL-6. Previous study has reported that GA protects HepG2 cells against TP-induced oxidative stress through Nrf2 pathway [22]. Thus, low-dose GA (50 mg/kg) may protect TP-induced oxidative stress and inflammation via activating Nrf2 signaling. Further investigations are expected.

Besides, GA may accelerate metabolism of TP in rat liver and thus reduce its hepatotoxicity. GA has previously been reported to be a CYP3A inducer when coadministrated with TP in rats [42], whereas TP is mainly metabolized by CYP3A of the cytochrome P450 superfamily [43]. Thus, CYP3A inducer can shorten the half-life (*t*_1/2_) of TP and reduce its hepatotoxicity, while CYP3A inhibitor has an opposite effect [43]. So another possible mechanism underlying the protection of GA against TP-induced hepatotoxicity may be that GA accelerated the metabolism of TP via inducing CYP3A, which merits further examination.

However, our results showed that GA in high-dose treatment had no protective effect on TP-induced liver injury. One previous study had reported that GA could contradictorily either prevent or induce mitochondrial permeability transition, whose specific effects depend on its concentration in rat heart mitochondria. Actually, GA prevents oxidative stress below 7.5 *μ*M, whereas, above this concentration, GA induces oxidative stress and produces ROS [44]. Similarly, Ishikawa et al. have reported that low-dose GL (less than 50 pM), the precursor substance of GA, inhibits the phosphorylation of protein kinase, but high-dose GL (more than 200 pM) has no such effect, indicating that the pharmacological activity of GL might depend on its concentration [45]. Although the discrepancy between the low and high doses of GA on hepatoprotection is reported for the first time, our results strongly recommended that the hepatic protective effect of GA on TP-induced liver injury is depending on its dose. This observation seems consistent with previously contradictory activities of high and low doses of GA/GL.

In previous literatures, GA exhibits hepatic protection at 5~75 mg/kg on rats (the equivalent dose for human is 0.79~11.9 mg/kg) [11, 20, 23, 24]. Our study demonstrated low-dose GA (50 mg/kg for rats, equivalent to 7.94 mg/kg for human) exerted a protective effect against TP-induced hepatotoxicity, but when given in a higher dose of 100 mg/kg (equivalent to 15.87 mg/kg for human), the protective function vanished. Nevertheless, it is inappropriate to simply extrapolate these results to human being. However, our observation together with previous studies warns us that the doses of GA should be carefully considered in clinical practice [44, 45]. Further studies are under way to investigate the dose-effect relationship of GA and underlying mechanisms.

## 5. Conclusions

Our study demonstrated the protection of low-dose GA (50 mg/kg) against TP-induced hepatotoxicity, which may mediate via anti-inflammation, antioxidation, and antiapoptosis. The protective effect is dose-related, which is only mediated by low-dose GA (50 mg/kg), but not high-dose GA (100 mg/kg). In addition, this study provides a notable evidence that the doses of GA/Licorice should be carefully considered when used together with TWHF or TWHF preparations.

## Figures and Tables

**Table 1:** Groups and treatments.

Group	Distilled water (*p.o.*, days 1~6)	GA (*p.o.*, days 1~6)	0.5% CMC-Na (*p.o.*, days 4~6)	TP (*p.o.*, days 4~6)
NC	+	—	+	—
GAH	—	100 mg/kg	—	—
TP	+	—	—	2.4 mg/kg
GAL+TP	—	50 mg/kg	—	2.4 mg/kg
GAH+TP	—	100 mg/kg	—	2.4 mg/kg

## Abbreviations

TP: Triptolide
TWHF: * Tripterygium wilfordii *Hook F
GL: Glycyrrhizin
GA: 18*β*-Glycyrrhetinic acid
ALT: Alanine aminotransferase
AST: Aspartate aminotransferase
ALP: Alkaline phosphatase
H&E: Hematoxylin and eosin
ROS: Reactive oxygen species
MDA: Malondialdehyde
SOD: Superoxide dismutase
GSH-Px: Glutathione peroxidase
CAT: Catalase
NF-*κ*B: Nuclear factor-kappa B
TUNEL: Terminal-deoxynucleotidyl transferase mediated nick end labeling
IHC: Immunohistochemical
DAB: Diaminobenzidine.
